# Supplementary material for: Smc5/6 functions with Sgs1-Top3-Rmi1 to complete chromosome replication at natural pause sites
Source: Nat Commun. 2021 Apr 8;12:2111. doi: 10.1038/s41467-021-22217-w (PMC8032827; doi:10.1038/s41467-021-22217-w)
Supplement: Supplementary file 1 — Supplementary Information [file 41467_2021_22217_MOESM1_ESM.pdf]

SUPPLEMENTARY INFORMATION

Smc5/6 functions with Sgs1-Top3-Rmi1 to complete chromosome replication at natural pause sites

Sumedha Agashe, **Chinnu Rose Joseph**, **Teresa Anne Clarisse Reyes, Demis Menolfi, Michele Giannattasio, Anja Waizenegger, Barnabas Szakal and**

Dana Branzei*

* Corresponding Author: Dana Branzei (dana.branzei@ifom.eu)

Supplementary Figures and Figure Legends

Supplementary Figure 1

Agashe et al

a

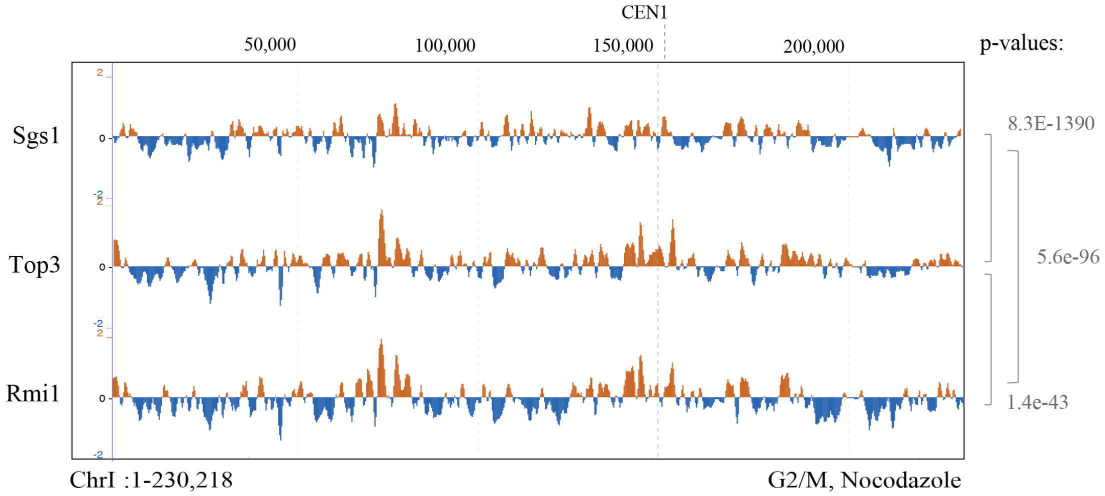

b

tRNA		
Protein	Fold change	p-value
Sgs1	1.18 (decrease)	0.067
Top3	2.15	1.9E-53
Rmi1	2.49	1.6E-69

c

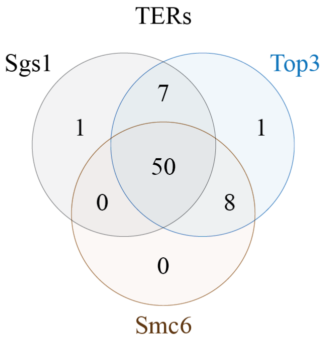

d

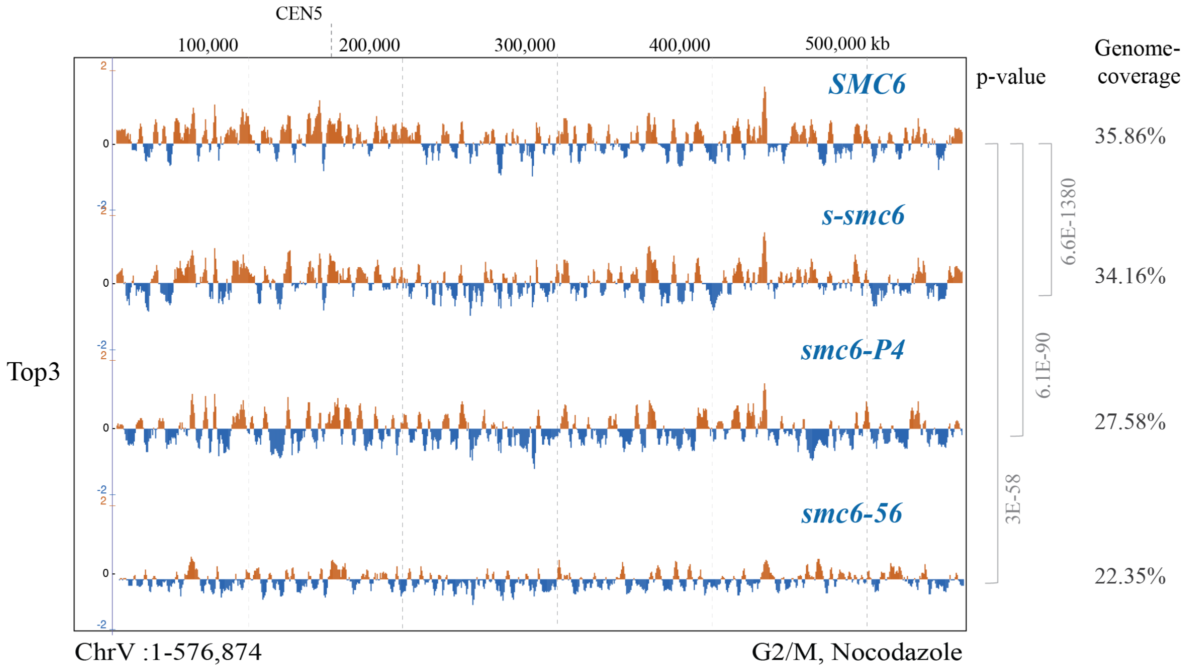

Supplementary Fig. 1. Top3 recruitment to chromatin is independent of Smc5/6, Related to Fig. 1. (a) ChIP-on-chip profile of HA-Sgs1, Top3-Flag, and Rmi1-Flag from G2/M-synchronized cells. Chr I is shown as example. The indicated p-values (one-tailed Fisher's exact test) relate to the genome-wide overlap between the considered protein clusters. Evaluation of the significance of overlap between the binding clusters of different proteins was performed by confrontation against a null hypothesis model generated with a Montecarlo-like simulation where the "score" for both the randomized positions and the actual data was calculated as the total number of overlapping bases among the whole clusters. The significance of correlation was scored using a one-tailed Fisher's exact test, as described in the Method section. (b) Analysis of overlap Sgs1, Top3 and Rmi1 at tRNAs genes. The table reports the fold increase/decrease of each protein at tRNA genes, calculated versus the ones expected for random binding, and the p-values (one-tailed Fisher's exact test) of the significance (see panel a). The values for Top3 and Rmi1 are also shown in Fig. 1b. (c) Smc6, Top3 and Sgs1 are enriched at NPSs that serve as termination sites (TERs) in G2/M. Values of overlap and non-overlap between the protein clusters are shown. (d) ChIP-on-chip profile of Top3-Flag from G2/M-synchronized WT, *s-smc6*, *smc6-P4* and *smc6-56* mutant cells. Chr V is shown as example. The indicated p-values (one-tailed Fisher's exact test) relate to the genome-wide overlap between the considered protein clusters (see legend of Fig. 1a for details). Genome-wide coverage in percentage of Top3 in different backgrounds is shown. Source data are provided as a Source Data file.

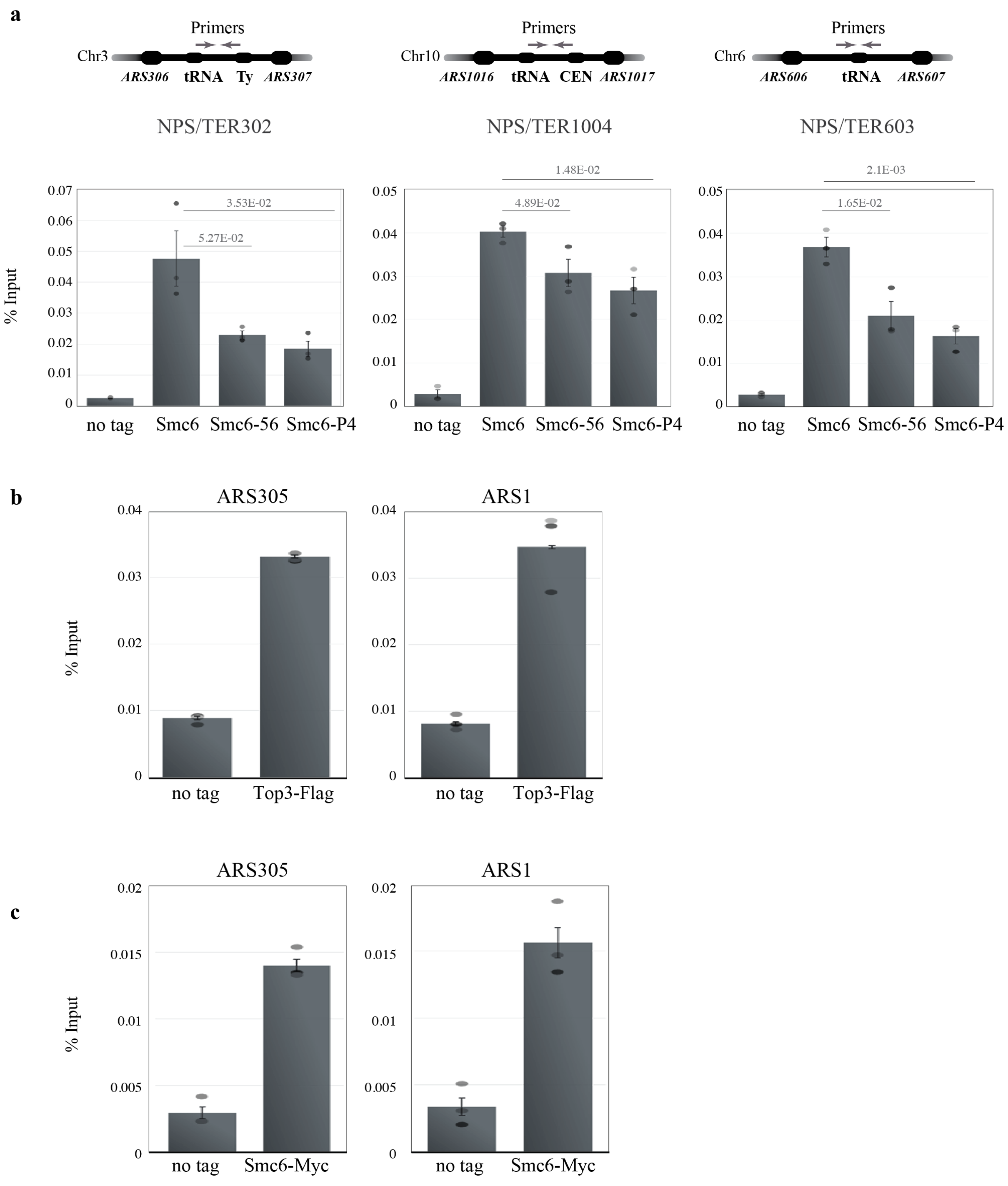

Supplementary Fig. 2. Smc5/6 and Top3 are recruited to natural pause sites and stalled replication forks, Related to Fig. 1. (a) ChIP-qPCR analysis of Myc-tagged Smc6 and variants versus no tag in G2/M phase at three indicated NPSs. (b, c) ChIP-qPCR analysis of Top3-Flag versus no tag (b) and Smc6-Myc versus no tag at stalled replication forks. Two early efficient

ARS regions (ARS305 and ARS1) were analyzed. (a-c) The mean of % input is derived from 3 biological replicates. Data are presented as mean values +/-SEM. p-values were calculated by unpaired two-sided t-test. Source data are provided as a Source Data file.

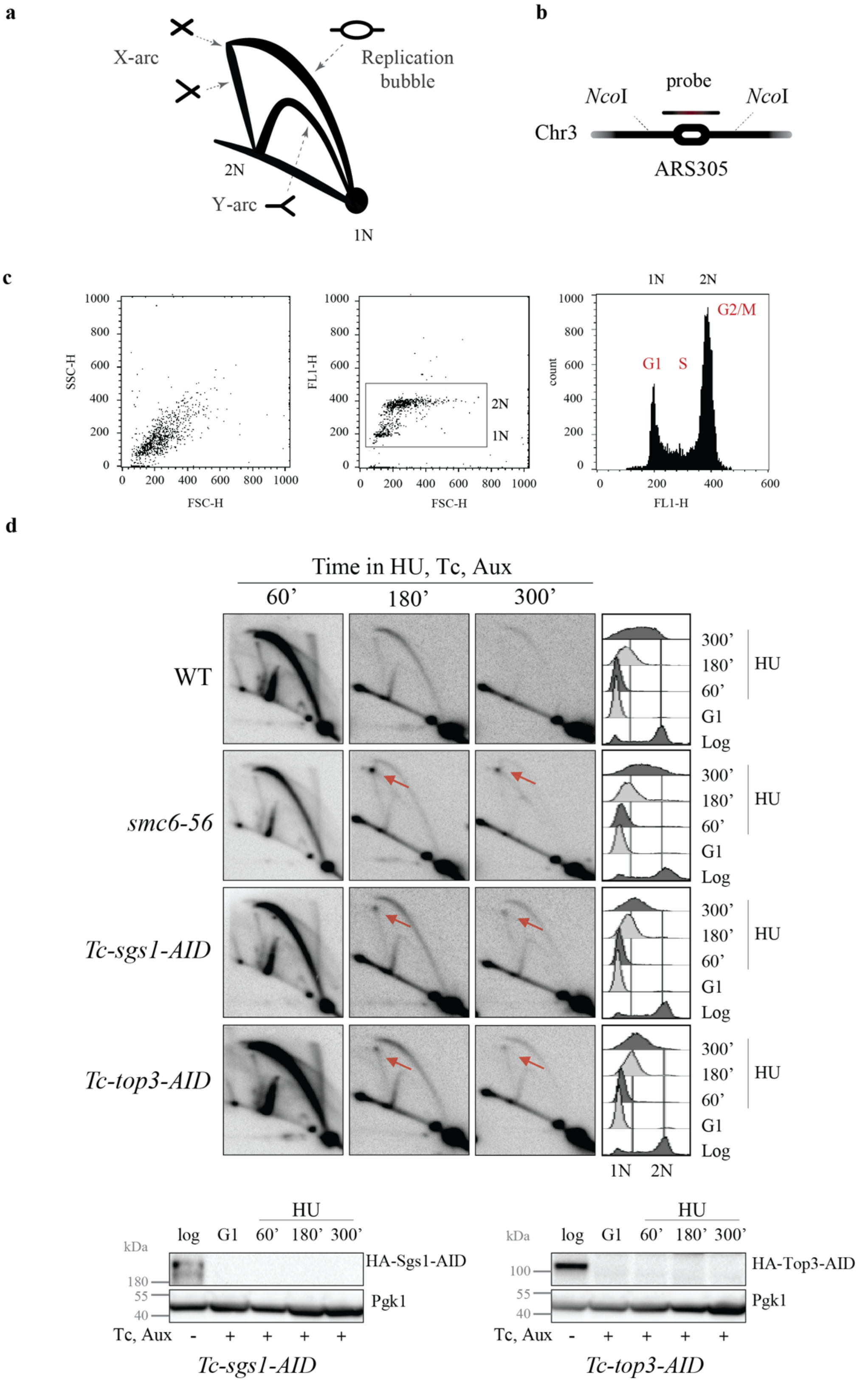
a
X-arc
Replication bubble
2N
Y-arc
1N
b
probe
NcoI
NcoI
Chr3
ARS305
c
SSC-H
FSC-H
FL1-H
FSC-H
2N
1N
count
FL1-H
1N
2N
G2/M
G1
S
d
Time in HU, Tc, Aux
60'
180'
300'
WT
smc6-56
Tc-sgs1-AID
Tc-top3-AID
300'
180'
60'
G1
Log
HU
1N
2N
HU
log
G1
60'
180'
300'
kDa
HA-Sgs1-AID
Pgk1
Tc, Aux
Tc-sgs1-AID
HA-Top3-AID
Tc-top3-AID

Supplementary Fig. 3. STR and Smc5/6 prevent joint molecule accumulation at stalled replication forks, Related to Fig. 2. (a) Schematic representation of replication intermediates observed by 2D gel electrophoresis at an early replication origin. (b) Schematic representation of the *ARS305* early replication origin analysed by 2D gel. (c) Flow Cytometry gating strategy and representative image. For all experiments in which cell cycle progression was followed, samples were gated on SSC-H and FSC-H as well as on FL1-H and FSC-H to exclude doublets and debris. Then a histogram of FL1-H values was generated from the remaining cells. A value of 200 in FL1-H represents the G1 population with a 1N DNA content and a value of 400 represents the G2 population with a 2N DNA content. 50.000 cells per sample were analysed. (d) Visualization of replication intermediates by 2D gel electrophoresis from cells of the indicated genotype at the ARS305 region. The cells were synchronized in G1 phase and released in media containing 200 mM HU, as well as Tetracycline (Tc) and Auxin (Aux). The experiment was repeated independently twice with similar results. Flow cytometry profiles are indicated on the right. Cells were collected at the indicated time-points. Sgs1 and Top3 tagged with HA were depleted with Auxin and Tetracycline, and depletion was confirmed by Western blotting. Pgk1 was used as loading control. Joint Molecules accumulating on the X-arc are indicated by arrows. Source data are provided as a Source Data file.

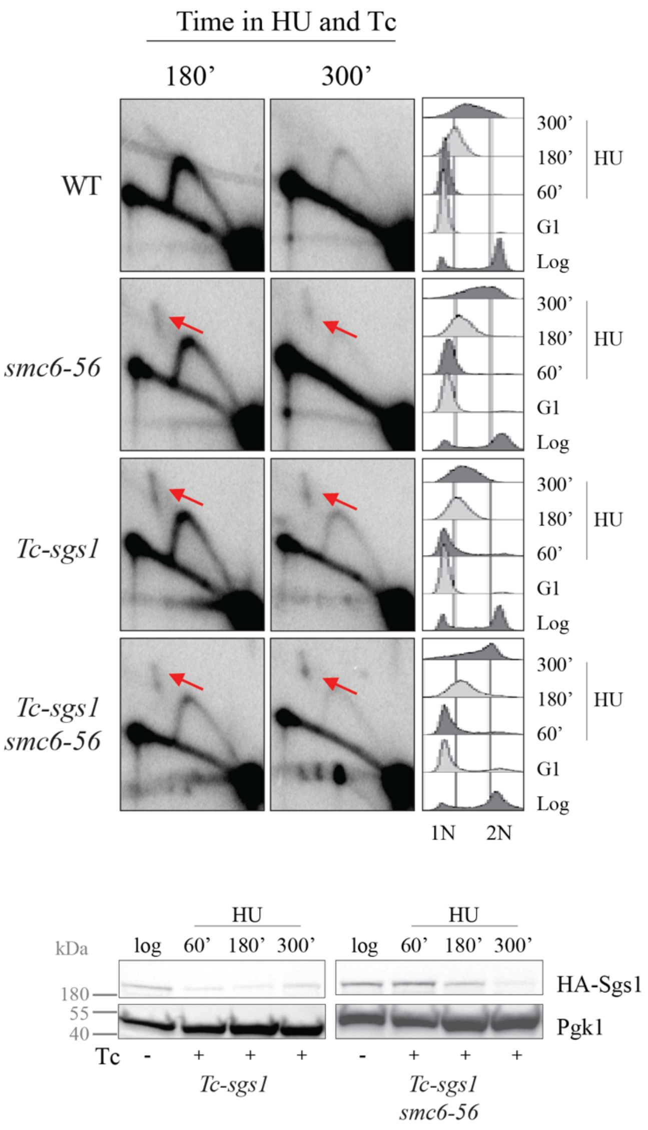

Supplementary Fig. 4. STR and Smc5/6 jointly prevent recombination intermediate accumulation at natural pause sites, Related to Fig. 2. (a, b) Visualization of replication intermediates arising at TER302 by 2D gel electrophoresis from cells of the indicated genotype synchronously released from G1 in media containing 200 mM HU, as well as Auxin (Aux) and Tetracycline (Tc). The experiment was repeated independently twice with similar results. FACS profiles indicated on the right show cell cycle progression. Sgs1 and Top3 tagged with

HA were depleted with Auxin and Tetracycline as indicated by WBs. Pgk1 was used as loading control. Joint molecules accumulating on the X-arc are indicated by arrows.

Source data are provided as a Source Data file.

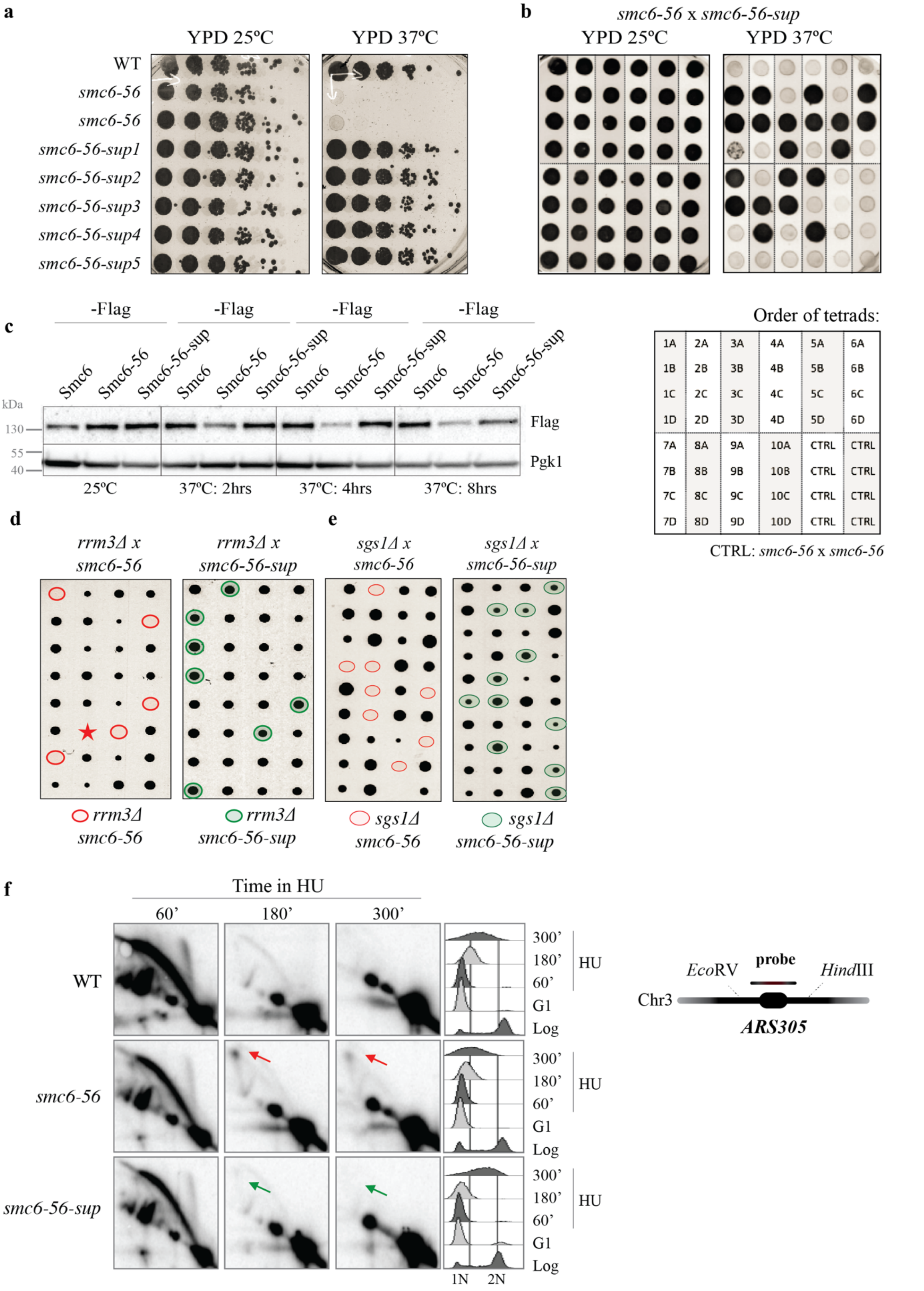

Order of tetrads:

1A	2A	3A	4A	5A	6A
1B	2B	3B	4B	5B	6B
1C	2C	3C	4C	5C	6C
1D	2D	3D	4D	5D	6D
7A	8A	9A	10A	CTRL	CTRL
7B	8B	9B	10B	CTRL	CTRL
7C	8C	9C	10C	CTRL	CTRL
7D	8D	9D	10D	CTRL	CTRL

CTRL: *smc6-56* x *smc6-56*

Supplementary Fig. 5. Validation and characterization of the *smc6-56* suppressor, Related to Fig. 4. (a) Rescue of the temperature sensitivity of *smc6-56* to WT level by suppressor mutation as observed by spot assay. (b) Validation of suppressor by checking 2:2 segregation of its temperature sensitivity when back-crossed with *smc6-56*, with separated tetrads spotted on the plates. (c) Smc6, Smc6-56 and Smc6-56-sup protein stability at 37ºC as assessed by Western blot. Pgk1 stands for loading control. The experiment was repeated independently three times with similar results. (d, e) Genetic interactions between *smc6-56* or *smc6-56-sup* with *rrm3Δ* and *sgs1Δ* checked by crossing with indicated genotype, segregation and marking the genotypes of the spores. Note additional lethality in the *smc6-56* cross with *rrm3Δ* marked with a star. (f) 2D gel electrophoresis of replication intermediates forming at *ARS305* in WT, *smc6-56*, *smc6-56-sup* strains. The cells were synchronized in G1 phase and released in media containing 200 mM HU. Flow cytometry profiles on the right show cell cycle progression. Joint molecules accumulating on the X-arc are indicated by arrows. Source data are provided as a Source Data file.

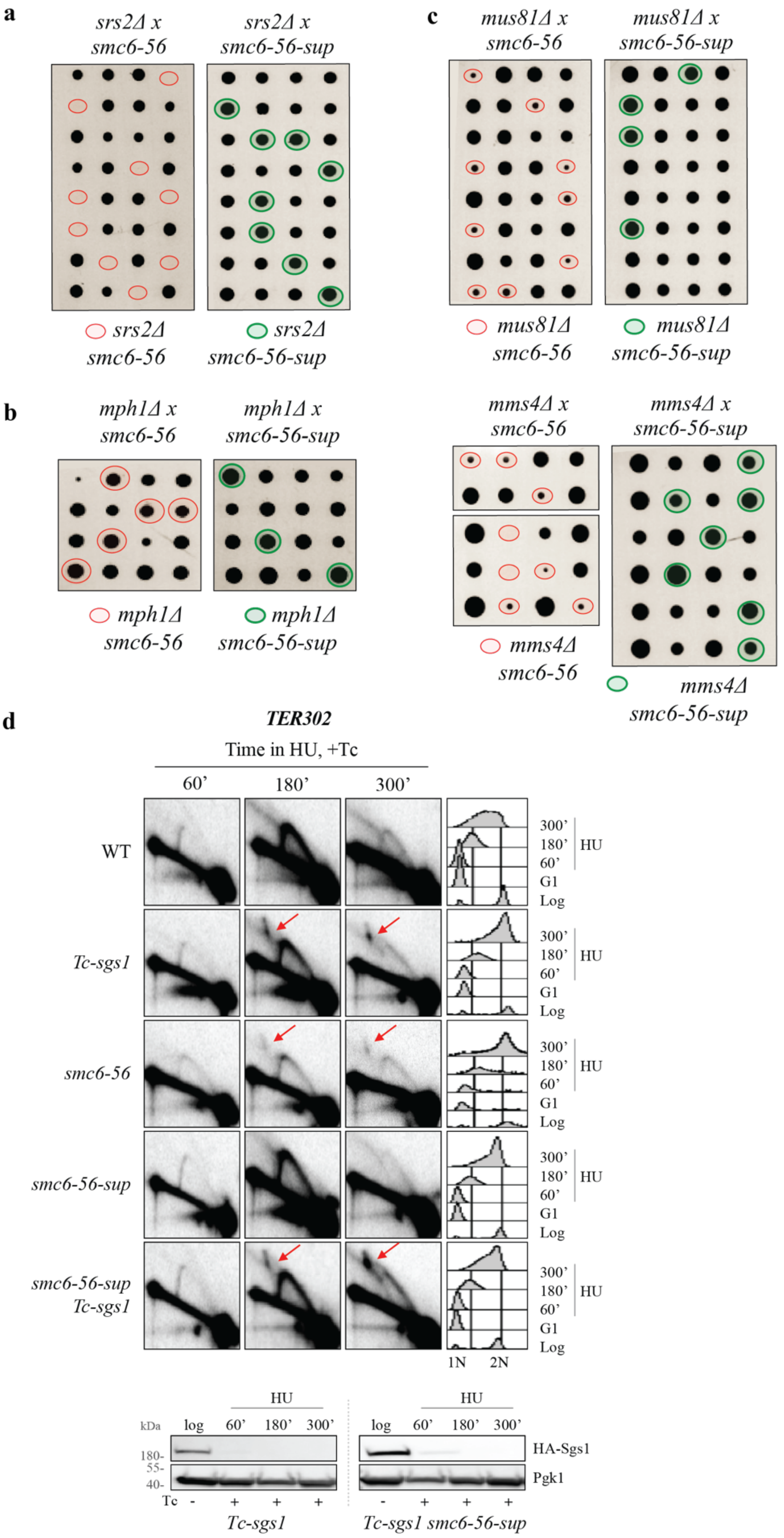
a
srs2Δ x smc6-56
srs2Δ x smc6-56-sup
srs2Δ smc6-56
srs2Δ smc6-56-sup
c
mus81Δ x smc6-56
mus81Δ x smc6-56-sup
mus81Δ smc6-56
mus81Δ smc6-56-sup
b
mph1Δ x smc6-56
mph1Δ x smc6-56-sup
mph1Δ smc6-56
mph1Δ smc6-56-sup
mms4Δ x smc6-56
mms4Δ x smc6-56-sup
mms4Δ smc6-56
mms4Δ smc6-56-sup
d
TER302
Time in HU, +Tc
60’ 180’ 300’
WT
Tc-sgs1
smc6-56
smc6-56-sup
smc6-56-sup Tc-sgs1
300’ 180’ 60’ HU G1 Log
1N 2N
HU
kDa log 60’ 180’ 300’
180-
55-
40-
Tc - + + +
HA-Sgs1
Pgk1
Tc-sgs1
Tc-sgs1 smc6-56-sup

Supplementary Fig. 6. *smc6-56-sup* genetic interactions with mutations in DNA helicases and resolvases, Related to Fig. 5. (a, b, c) Genetic interactions between *smc6-56* or *smc6-56-sup* with *srs2*Δ*, mph1*Δ*, mus81*Δ and *mms4*Δ as checked by crossing. (d) Visualization of replication intermediates forming proximal to *TER302* by 2D gel electrophoresis from cells of the indicated genotype, synchronized in G1 and released in media containing 200 mM HU and Tetracycline (Tc). The experiment was repeated independently twice with similar results. Flow cytometry profiles are shown on the right. Sgs1, tagged with HA, was depleted with Tetracycline as verified by Western blotting. Pgk1 was used as loading control. Joint molecules (JMs) accumulating on the X-arc are indicated by arrows. Source data are provided as a Source Data file.

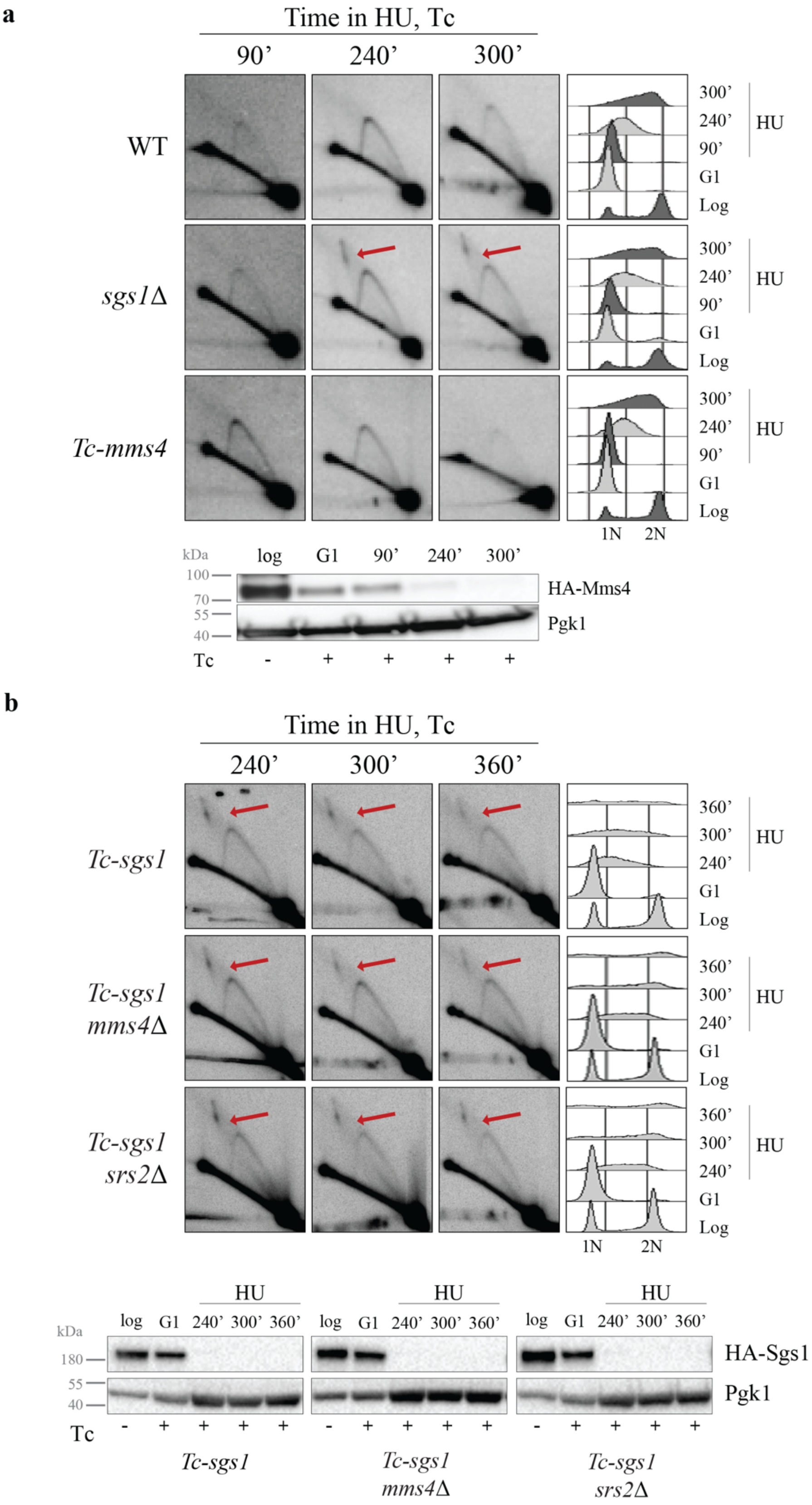
a
Time in HU, Tc
90’
240’
300’
WT
sgs1Δ
Tc-mms4
300’
240’
90’
G1
Log
HU
1N
2N
kDa
log
G1
90’
240’
300’
100
70
55
40
HA-Mms4
Pgk1
Tc
-
+
+
+
+
b
Time in HU, Tc
240’
300’
360’
Tc-sgs1
Tc-sgs1 mms4Δ
Tc-sgs1 srs2Δ
360’
300’
240’
G1
Log
HU
1N
2N
HU
log
G1
240’
300’
360’
kDa
180
55
40
HA-Sgs1
Pgk1
Tc
Tc-sgs1
Tc-sgs1 mms4Δ
Tc-sgs1 srs2Δ

Supplementary Fig. 7. Mus81-Mms4 and Srs2 mutations do not increase joint molecules at natural pause sites, Related to Fig. 7. (a, b) Visualization of replication intermediates forming proximal to *TER302* by 2D gel electrophoresis from cells of the indicated genotype, synchronized in G1 and released in media containing 200 mM HU (a) or 150 mM HU (b) and Tetracycline (Tc). The experiments were repeated independently twice with similar results. Flow cytometry profiles are shown on the right. Mms4 and Sgs1 tagged with HA, were depleted with Tetracycline as verified by Western blotting. Pgk1 was used as loading control. Source data are provided as a Source Data file.

Supplemental Items

Supplementary Table 1. *Saccharomyces cerevisiae* Strains Used in This Study.

Strain name	genotype	Source
FY1296	*Mat A ade2-1 trp1-1 leu2-3 112 his3-11 15 ura3 can1-100 Rad5+ (W303)*	Lab collection
FY1646	*Mat alpha ade2-1 trp1-1 leu2-3 112 his3-11 15 ura3 can1-100 Rad5+ (W303)*	Lab collection
FY1332	*W303 Mat A smc6-P4-13MYC::KANMX4*	Lab collection
FY1432	*W303 Mat A smc6-56-13MYC::KANMX4*	Lab collection
HY1729	*W303 Mat alpha mms4Δ::HPHMX4*	Lab collection
FY1744	*W303 Mat A sgs1-sim::KANMX*	Lab collection
FY1746	*W303 Mat A sgs1-K621R::KANMX*	Lab collection
FY1060	*W303 Mat A sgs1Δ::HIS3MX6*	Lab collection
HY2806	*W303 Mat A SMC6-6HIS-3FLAG::KANMX4*	Lab collection
HY3167	*W303 Mat A S::NATNT2-SMC6*	Lab collection
HY3293	*W303 Mat A pADH1-tc3-3xHA-Top3::NATMX4*	Lab collection
HY3611	*W303 Mat A rmi1Δ::KANMX4*	Lab collection
HY3674	*W303 Mat A ura3-1::ADH1-OsTIR1-9MYC(URA3) top3::pADH1-tc3-3xHA-Top3(HPHMX4)-AID::NATMX4*	Lab collection
HY3707	*W303 MATa RAD5+, sgs1::pADH1-tc3-6xHA-Sgs1 (KanMX4)*	Lab collection
HY3721	*W303 Mat A ura3-1::ADH1-OsTIR1-9Myc(URA3), sgs1::pADH1-tc3-3xHA(HPHMX4)-Sgs1-aid(KANMX4)*	Lab collection
HY3807	*W303 Mat A TOP3-6HIS-10FLAG::KANMX4*	Lab collection
HY3882	*W303 Mat A sgs1::pADH1-tc3-3xHA-Sgs1 (NATMX)*	Lab collection
HY4071	*W303 Mat A rrm3Δ::HIS3MX6*	Lab collection
HY4898	*W303 Mat A S::NATNT2-Mms21*	Lab collection
HY4916	*W303 Mat A Rrm3-10FLAG::KANMX4*	Lab collection
HY4921	*W303 Mat A sgs1::pADH1-tc3-3xHA-Sgs1(NATMX) mms4Δ::HPHMX*	Lab collection
HY6606	*W303 Mat A S::NATNT2-Mms21-PK9-HIS3MX6*	Lab collection
HY7390	*W303 Mat A mms4::pADH1-tc3-3xHA-Mms4::HPHMX*	Lab collection
HY7512	*W303 Mat alpha MMS4-6HIS-3FLAG::KANMX4*	Lab collection
HY7717	*W303 Mat A SMC6-13myc::TRP*	This study
HY7937	*W303 Mat A smc6-P4-13myc::KANMX TOP3-6HIS-10FLAG::KANMX4*	This study
HY8031	*W303 Mat A S::NATNT2-SMC6 TOP3-6HIS-10FLAG::KANMX4*	This study
HY8032	*W303 Mat A smc6-56-13myc::KANMX TOP3-6HIS-10FLAG::KANMX4*	This study
HY8112	*W303 Mat A Rmi1-6HIS-10FLAG::KANMX4*	This study
HY8288	*W303 Mat A smc6-56-FLAG::KANMX4*	This study
HY8455	*W303 Mat alpha srs2Δ::HIS3MX6*	Lab collection

HY8767	*W303 Mat alpha mph1Δ::HPHMX4*	Lab collection
HY8947	*W303 Mat A pADH1-tc3-3xHA-Top3::NATMX4 S::NATNT2-SMC6*	This study
HY9342	*W303 Mat A sgs1-K621R::KANMX TOP3-6HIS-10FLAG::KANMX4*	This study
HY9387	*W303 Mat A Smc6-56-Sup-13MYC::KANMX4 TOP3-6HIS-10FLAG::KANMX4*	This study
HY9390	*W303 Mat A Smc6-56-Sup-13MYC::KANMX4*	This study
HY9410	*W303 Mat A mms21-CH::HIS TOP3-6HIS-10FLAG::KANMX4*	This study
HY9864	*W303 Mat alpha mus81Δ::NATMX4*	Lab collection
HY9883	*W303 Mat A sgs1::pADH1-tc3-3xHA-Sgs1(NATMX) srs2Δ::HIS3MX6*	Lab collection
HY10146	*W303 Mat A sgs1::pADH1-tc3-3xHA-Sgs1(HPHMX4) smc6-56-Sup-13MYC::KANMX4*	This study
HY10149	*W303 Mat A sgs1::pADH1-tc3-3xHA-Sgs1(HPHMX4) smc6-56-13MYC::KANMX4*	This study
HY10448	*W303 Mat A sgs1Δ::NATMX4 Smc6-56-Sup-13MYC::KANMX4*	This study
HY10490	*W303 Mat A mus81Δ::NATMX4 Smc6-56-13MYC::KANMX4*	This study
HY10491	*W303 Mat A mus81Δ::NATMX4 Smc6-56-Sup-13MYC::KANMX4*	This study
HY10492	*W303 Mat A mms4Δ::HPHMX4 Smc6-56-13MYC::KANMX4*	This study
HY10493	*W303 Mat A mms4Δ::HPHMX4 Smc6-56-Sup-13MYC::KANMX4*	This study
HY10494	*W303 Mat A mph1Δ::HPHMX4 Smc6-56-13MYC::KANMX4*	This study
HY10496	*W303 Mat A mph1Δ::HPHMX4 Smc6-56-Sup-13MYC::KANMX4*	This study
HY10498	*W303 Mat A ura3-1::ADH1-OsTIR1-9MYC(URA3) top3::pADH1-tc3-3xHA-Top3(HPHMX4)-AID::NATMX4 Smc6-56-Sup-13MYC::KANMX4*	This study
HY10501	*W303 Mat A ura3-1::ADH1-OsTIR1-9MYC(URA3) top3::pADH1-tc3-3xHA-Top3(HPHMX4)-AID::NATMX4 Smc6-56-13MYC::KANMX4*	This study
HY10633	*W303 Mat A srs2Δ::HIS3MX6 Smc6-56-Sup-13MYC::KANMX4*	This study
HY10800	*W303 Mat A mms4::pADH1-tc3-3xHA-Mms4-10FLAG(KANMX)::HPHMX*	Lab collection
HY10843	*W303 Mat A smc6-56-sup-13myc::KANMX sgs1-sim::KANMX*	This study
HY10845	*W303 Mat A smc6-56-sup-13myc::KANMX sgs1-K621R::KANMX*	This study
HY10984	*W303 Mat alpha smc6-56-13myc::KANMX MMS4-6HIS-3FLAG::KANMX4*	This study
HY10986	*W303 Mat alpha smc6-56-sup-13myc::KANMX MMS4-6HIS-3FLAG::KANMX4*	This study
HY11551	*W303 Mat A smc6-P4-FLAG::KANMX4*	This study
HY11575	*W303 Mat A Smc6-56-Sup-13MYC::KANMX4 (de novo created)*	This study

Supplementary Table 2. List of oligos used for qPCR

Oligo	purpose	Sequence
TER302CF	q-PCR primer at TER302	5'-GGGTAGACGAAACTATA TACGCAAT-3'
TER302CR	q-PCR primer at TER302	5'-TGCCCTCCTCCTTGTCAATA-3'
TER603AF	q-PCR primer at TER603	5'-ATGGGGGTTGAACATTGTGT-3'
TER603AR	q-PCR primer at TER603	5'-TCGCATATAAGCAAGTGGTTT-3'
TER1004FF	q-PCR primer at TER1004	5'-CCATCTTGTTGTCCATGTCC-3'
TER1004FR	q-PCR primer at TER1004	5'-CGCATGGGATTTTGCTATC-3'

Supplementary Table 3. List of oligos used for 2D gel probes

Oligo	purpose	Sequence
TER302Fw	amplification of termination probe (2D)	5'-GAAGGTTCAACATCAATTGATTG ATTCTGCCGCCATGATC-3'
TER302Rv	amplification of termination probe (2D)	5'-GCTTCCCTAGAACCTTCTTATGTT TTACATGCGCTGGGTA-3'
ARS305FW	amplification of ARS305 probe	5'-GTTCCGAAACAGGACACTTAGC-3'
ARS305RV	amplification of ARS305 probe	5'-ATCCAGGAGGGACTCAATGTAG-3'
